# Autophagy and Autoimmunity Crosstalks

**DOI:** 10.3389/fimmu.2013.00088

**Published:** 2013-04-15

**Authors:** Abhisek Bhattacharya, N. Tony Eissa

**Affiliations:** ^1^Department of Medicine, Baylor College of MedicineHouston, TX, USA

**Keywords:** autophagy, autophagosome, autoimmunity, encephalomyelitis, autoimmune, experimental, lupus erythematosus, systemic

## Abstract

Autophagy, initially viewed as a conserved bulk-degradation mechanism, has emerged as a central player in a multitude of immune functions. Autophagy is important in host defense against intracellular and extracellular pathogens, metabolic syndromes, immune cell homeostasis, antigen processing and presentation, and maintenance of tolerance. The observation that the above processes are implicated in triggering or exacerbating autoimmunity raises the possibility that autophagy is involved in mediating autoimmune processes, either directly or as a consequence of innate or adaptive functions mediated by the pathway. Genome-wide association studies have shown association between single nucleotide polymorphisms (SNPs) in autophagy related gene 5 (*Atg5)*, and *Atg16l1* with susceptibility to systemic lupus erythematosus (SLE) and Crohn’s disease, respectively. Enhanced expression of *Atg5* was also reported in blood of mice with experimental autoimmune encephalomyelitis (EAE), a mouse model of multiple sclerosis (MS), and in T cells isolated from blood or brain tissues from patients with active relapse of MS. This review explores the roles of autophagy pathway in the innate and adaptive immune systems on regulating or mediating the onset, progression, or exacerbation of autoimmune processes.

*The autophagy* pathway, an evolutionary conserved mechanism, starts with the development of an isolation membrane within the cell that engulfs damaged organelles, misfolded proteins or pathogens, and eventually develops into an autophagosome. The autophagosomes, in turn, fuse with lysosomes to form the autophagolysosomes where the actual degradation of the substrates takes place (Levine et al., 2011). For the individual cell, the autophagy pathway is important not only to get rid of foreign or unwanted materials but also for efficient energy recycling during periods of stress. For the whole organism, the immune and physiological consequences of aberration of the autophagy pathway are much more profound. The immune system, responsible for surveillance and communication between different organs and cells types, is one such system in which the role of autophagy and the consequences of defects in autophagy go far beyond the degradative role of the pathway (Deretic, 2012a). Figure 1 shows potential roles of the autophagy pathway in the adaptive and innate immune systems that might modulate the onset and outcome of an autoimmune disease.

**Figure 1:**
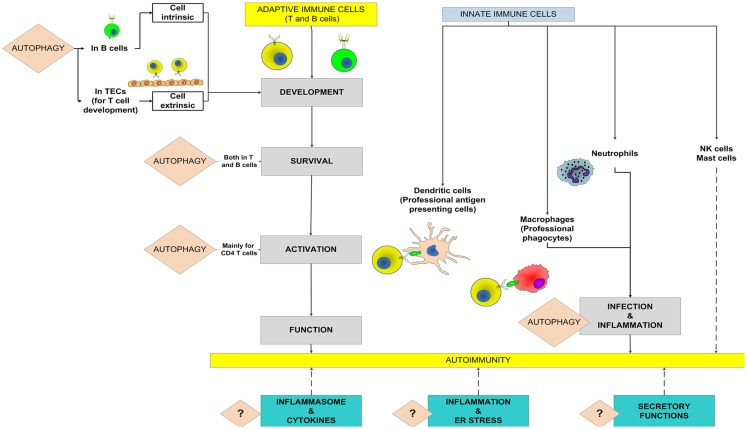
**Potential roles of autophagy in the adaptive and innate immune systems to mediate autoimmunity**. The known roles of autophagy in the contributing processes are highlighted and the broken lines show potential contributions toward autoimmunity. A question mark denotes the possibility that the autophagy pathway might modulate autoimmune diseases through these processes. TECs, thymic epithelial cells.

## Autophagy, the Adaptive Immune System and Autoimmunity

Autophagy plays important roles in both innate and adaptive immunity. Because there have been several excellent reviews on this topic (Munz, 2009; Sumpter and Levine, 2010; Kuballa et al., 2012; Randow and Munz, 2012), we will only discuss brief aspects of these roles as they might pertain to autoimmunity. Autophagy is essential for survival and homeostasis of lymphocytes and there exist at least two broad stages where autophagy might affect the adaptive immune cells. As the development of lymphocyte is a complex process involving inputs from other cells, both lymphocyte-intrinsic and extrinsic defects in autophagy might affect development and/or maturation of lymphocytes.

## Autophagy in Lymphocyte Development

T cell development in the thymus undergoes positive and negative selections, processes in which extrinsic inputs from thymic epithelial cells (TECs) play a major role in shaping the T cell repertoire. TECs show high levels of constitutive autophagy essential for proper display of MHC-antigen complex on their surface (Mizushima et al., 2004; Kasai et al., 2009), thereby facilitating appropriate T cell selection. Mice with *Atg5* deficiency in TECs showed severely impaired central tolerance and autoimmune organ destruction, suggesting that autophagy-mediated display of MHC-antigen complex on surface of TECs is essential for proper T cell development (Nedjic et al., 2008). Autophagy deficiency in the TECs impaired both positive and negative selection mechanisms resulting into autoimmunity and it was proposed that autophagy-dependent display in the peripheral tissue needed to be counterbalanced by a similar tolerogenic mechanism in the thymus in order to prevent such autoimmune processes (Nedjic et al., 2008). Further, a recent report demonstrated the requirement of autophagy in TECs for loading endogenous antigens onto MHC-II and that this process was essential for negative selections of CD4 T cells (Aichinger et al., 2013). Because both DCs and TECs might be important in differentiation of regulatory T cells (Tregs) (Wirnsberger et al., 2009; Hinterberger et al., 2010), this report suggested that autophagy might be important in differentiation of Tregs (Aichinger et al., 2013). As Tregs are among the major players controlling autoimmunity (La Cava, 2009), this might be another potential link between autophagy and autoimmune diseases.

Fetal liver chimera and conditional knock-out studies have shown that T cell development remained normal in mice lacking *Atg5* in T cells but peripheral T cell compartment showed reduction in numbers, particularly in CD8 T cells (Pua et al., 2007). These results were attributed to the pro-survival role of autophagy in mature T cells. Studies showed considerable interaction between the autophagy and apoptotic pathways (Maiuri et al., 2007). *Atg3*, *5*, *or* *7*-deficient mature T cells showed defective Endoplasmic reticulum (ER) homeostasis and mitochondrial clearance and, consequently, an elevated levels of ROS, which might serve as one of the potential links between the autophagy and apoptotic pathways (Pua et al., 2007; Jia and He, 2011; Jia et al., 2011). However, increased levels of mitochondria were observed in *Atg7−/−* but not in *Atg5−/−* thymocytes at the single positive stage (Pua et al., 2009). A possible explanation could be different stages or extent of involvement of these proteins in mitochondrial clearance. These findings potentially brings another layer of complexity into focus, namely autophagy-independent effect of various *Atg*.

In contrast to T cells, autophagy in B cells plays a very important role in development and the requirement of *Atg5* has been found to be highly stage-specific, with a defective pro- to pre-B cell transition in B cell-specific *Atg5−/−* knock-out mice. In these mice, the levels of pre and immature B cells, along with peritoneal B1 cells were reduced to a great extent. This finding was also attributed to a role of autophagy in maintaining B cell survival (Miller et al., 2008).

## Autophagy in Lymphocyte Functions

Autophagy induction in response to starvation and TCR stimulation has been observed in mouse T cells (Pua et al., 2007) and in cultured human T cells during aging (Gerland et al., 2004) and in HIV infection (Espert et al., 2006). *Atg5*-deficient T cells showed reduced proliferation upon both TCR and PMA-ionomycin stimulation (Pua et al., 2007). This finding highlights potentially different roles of autophagy in naïve versus activated T cells. Most studies involving autophagy in T cells focused on roles of autophagy in cell survival and found autophagy to be a pro-survival mechanism (Pua et al., 2007). However, some studies have also suggested that autophagy might be required for T cell death (Espert et al., 2006; Bell et al., 2008). Uninfected lymphocytes undergo autophagy-mediated cell death upon engagement of the receptor CXCR4 by HIV envelop glycoprotein (Espert et al., 2006). Another study has also shown that autophagy could be an important cell death machinery in T cells lacking caspase-8 or Fas-associated death domain (FADD) activity, thereby raising the possibility that interaction between autophagy and apoptosis might be context dependent (Bell et al., 2008). It is possible that, in activated T cells, autophagy plays different roles compared to naïve cells and might be involved in activation-induced cell death following T cell proliferation in immune response.

Recent findings suggest that autophagy might affect overall T cell functions under different conditions of polarization and activation. Rapamycin, an mTOR inhibitor and inducer of autophagy, has been found to promote T cell memory when administered in low doses, although it is not clear if this effect is mediated by autophagy (Araki et al., 2009). Moreover, low dose rapamycin exacerbated autoimmune experimental uveitis, and this action of rapamycin was thought to be mediated by autophagy (Zhang et al., 2012). Interestingly, expression of *Atg5* has been shown to correlate with severity of experimental autoimmune encephalitis (EAE), a mouse model of multiple sclerosis (MS), and to be increased in T cells of MS patients during relapses (Alirezaei et al., 2009), which can worsen by prolonged autoreactive T cell survival. EAE is considered predominantly a CD4 mediated disease and further studies are required to dissect how autophagy in T cells influences the onset or progression of autoimmune diseases in animal models and if these roles of autophagy are also dependent on cell survival. These studies can develop *in vivo* models in which the roles of autophagy in CD4 or CD8 cells could be studied independent to its pro- or anti-survival functions, particularly in the context of an infection or autoimmune disease.

In mature B cells, BCR signal can lead to B cell activation or apoptosis, depending on the context. Autophagy is involved in both processes, with BCR-activation-mediated cell death being associated with extensive autophagosome formation (Watanabe et al., 2008). B cells are capable of antigen processing following BCR ligation and autophagy might be involved in such process (Watanabe et al., 2008). BCR signaling recruits TLR-9 to autophagosome for further interaction with its ligand (Chaturvedi et al., 2008). Systemic lupus erythematosus (SLE) is perhaps the most studied autoimmune disease with respect to the roles of autophagy in autoimmune processes (Pierdominici et al., 2012). There are a number of potential mechanisms by which autophagy might influence the pathogenesis of SLE, modulating both the adaptive and innate immune system. As B cells represent a major player in SLE, in which they act by both antibody-dependent and antibody-independent mechanisms, autophagy-mediated B cell modulation might directly influence the pathophysiology of SLE.

## Autophagy, the Innate Immune System and Autoimmunity

Autoimmunity results from uncontrolled action of the adaptive immune cells, however, activation of the adaptive system depends on the innate immune cells and the innate immune system is perhaps the most extensively studied component with respect to the role of autophagy in shaping the organization and functions of the system (Deretic, 2012b).

The innate immune functions can be broadly categorized into four overlapping stages, migration, recognition and phagocytosis, antigen processing and presentation, and cytokine secretion. Autophagy plays particularly important roles in the last three stages, thereby not only shaping the innate immune response but also influencing the activation of the adaptive immune compartment.

## Level One: Phagocytosis, Autophagy, and Autoimmunity

The role of autophagy in innate immunity is best characterized with respect to pathogen elimination. Both pathogen recognition and intracellular killing can be controlled by autophagy. The autophagy pathway interacts extensively with a number of pattern recognition receptors (PRR) and PRR activation in a wide variety of cases has been shown to induce autophagy (Tang et al., 2012). However, recent evidences suggest that this process might also extend beyond pathogen control.

Phagocytosis can be viewed as a coordinated interaction between two different kinds of players, a predator that engulfs the materials to be cleared, macrophages being the professional phagocytes in the body, and prey to be engulfed such as a pathogen, foreign materials, or dead cells. Autophagy has been found to be an essential process for dead cell clearance. Apoptotic cells release lysophosphatidylcholine (LPC) as a “come-get-me” signal for phagocytes and upregulate phosphatidylserine (PS) as an “eat me” signal on their surface. Autophagy genes are essential for efficient release of LPC and in absence of autophagy, apoptotic cells fail to express PS properly on their surface (Qu et al., 2007).

On the other hand, proteins involved in autophagy pathway, such as LC3-II (Microtubule-associated protein 1 light chain 3 alpha), Beclin 1, and VPS34, are recruited to phagosomes following phagocytosis of particles containing TLR ligands by macrophages (Sanjuan et al., 2007). LC3-associated phagocytosis, a process distinct from classical autophagy, has also been found to be necessary to carry out efficient dead cell clearance (Martinez et al., 2011) and defects in expression of MARCO (macrophage receptor with collagenous structure), a receptor involved in dead cell clearance, has been shown to result into reduced dead cell clearance and SLE in mice (Rogers et al., 2009). Thus, an absence of autophagy or autophagic proteins might result into defective clearance of apoptotic cells. As defects in apoptotic cell clearance have been linked to a number of autoimmune diseases, such as SLE, it is possible that autophagy might modulate the susceptibility to autoimmunity.

It should also be noted that autophagy induction in macrophages has been shown to affect phagocytosis of pathogens, though the reports are conflicting, indicating both increase and decrease in phagocytosis following induction of autophagy (Martinet et al., 2009; Lima et al., 2011). A number of autoimmune diseases are precipitated or exacerbated following infection (Kivity et al., 2009). It would be important to determine if changes in apoptotic cell clearance occur following infection-induced modulation in autophagy, which in turn could modulate the induction or exacerbation of autoimmune processes.

## Level Two: Antigen Presentation, Autophagy, and Autoimmunity

The classic definition of antigen presentation is that extracellular antigens are presented in the context of class II MHC following endocytosis and phagolysosomal degradation (Gannage and Munz, 2010). Recent evidence suggests that this process depends on the autophagy pathway. Characterization of the MHC-II ligands, called ligandome, in a human B lymphoblastoid cell line showed that peptides from intracellular sources are presented on MHC-II and starvation-induced autophagy enhanced this process (Dengjel et al., 2005). Further, autophagosomes colocalize with MHC-II loading compartments in two important antigen presenting cells (APCs) that shape up the entire adaptive immune repertoire. These cells are TECs, that shape up the T cell repertoire, and the dendritic cells (DC) that act as the professional APCs (Schmid and Munz, 2007; Schmid et al., 2007). Mice with DCs lacking *Atg5* succumbed to HSV-2 infection and showed defective CD4 T cell priming. These DCs showed defective antigen presentation resulting from a profound defect in processing and delivery of antigens containing TLR ligands to MHC-II compartment and delayed phagolysosomal fusion and degradation of the antigens (Lee et al., 2010). Autophagy induction in bone-marrow DCs also enhanced presentation of mycobacterial antigen and mice immunized with rapamycin-treated DCs showed stronger T cell response upon challenge with *Mtb* (Jagannath et al., 2009). Autophagy in APCs is involved in presentation of citrullinated peptide, a hallmark of rheumatoid arthritis, in context of class II MHC (Ireland and Unanue, 2011). It also has been suggested that autophagy may be involved in class I antigen presentation to CD8 T cells, particularly in context of viral infection (English et al., 2009; Uhl et al., 2009).

Dendritic cell-mediated antigen presentation in the context of MHC-II is perhaps an area where the role of autophagy could directly influence autoimmune diseases. Activation of the adaptive immune cells, the major players in most autoimmune diseases, depends primarily on DC-mediated antigen presentation. Genome-wide association studies have identified *Atg5* as one of the susceptibility loci in SLE (Harley et al., 2008; Gateva et al., 2009; Han et al., 2009; Zhou et al., 2011a), though the functional significance of this finding is yet to be established. A number of possibilities have been raised ranging from increased survival of pathogenic T cells to defects in apoptotic cell clearance and several autophagy modulators are currently in clinical trials for SLE (Pierdominici et al., 2012). It is interesting to note that the *PRDM1-ATG5* intergenic region has also been associated with susceptibility to rheumatoid arthritis (Raychaudhuri et al., 2009) and a common role of the autophagy pathway in different autoimmune diseases has been proposed (Zhou et al., 2011a). Another autophagy gene associated with autoimmunity is *Atg16l1*, being implicated in Crohn’s disease (CD) (Parkes et al., 2007). DCs with patients of CD, harboring particular *Atg16l1* risk variant, showed defects in autophagy induction and in presentation and priming of pathogen-specific CD4 T cells (Cooney et al., 2010). Interaction between the gut microflora and the mucosal immune system plays a pivotal role in CD (Manichanh et al., 2012) and autophagy in mucosal immune cells might also influence the pathophysiology and outcome of CD. Indeed, a recent report showed that an intact autophagy pathway restricted intracellular replication of adherent-invasive *Escherichia coli*, implicated in the pathogenesis of CD; without affecting the replication of other commensal or pathogenic strains of *E. coli* involved in gastroenteritis (Lapaquette et al., 2010).

It would be informative to determine the phenotype of mice with autophagy deficiency in DCs, in autoimmune disease models of MS, a predominantly CD4 T cell mediated disease, or rheumatoid arthritis.

## Level Three: Cytokines, ER Stress, Autophagy, and Autoimmunity

The third important link between autophagy and autoimmunity could be through modulating cytokine secretion, particularly in the context of inflammasome activation. Autophagy plays a negative role with respect to inflammasome activation and autophagy deficiency leads to increased production of IL-1β and IL-18 (Nakahira et al., 2011; Zhou et al., 2011b). Diseases resulting from increased activation of the immune system comprise two different categories: autoinflammatory diseases, characterized by inflammation mediated predominantly by innate immune cells, including macrophages and neutrophils, and autoimmune diseases in which the adaptive immune cells target the self-antigens (McGonagle et al., 2009). The inflammasome-mediated effects belong to the former category and the role of inflammasomes in these diseases has been reviewed (Shaw et al., 2011). However, given the extensive effects of IL-1β on adaptive immune cells, autophagy might also affect the outcome of autoimmune diseases by modulating IL-1β production. As a whole, IL-1β and IL-18 enhance the functional responses of B and T cells including IL-2 receptor expression and lifespan, antibody production by B cells, and T_H_1 and T_H_17 polarization effects (Ben-Sasson et al., 2009; Chung et al., 2009). Thus IL-1β and IL-18 might well serve as a bridge between autophagy in innate cells and the adaptive immune response. In this context, IL-1β receptor blockade had beneficial effects in rheumatoid arthritis and has been suggested as a therapy for autoinflammatory diseases (Goldbach-Mansky, 2009). Conflicting reports exist regarding the role of inflammasome activation in EAE, with one study showing roles of NLRP3 inflammasome in EAE progression (Gris et al., 2010), whereas another study found no such role but reported an inflammasome-independent role of ASC (Shaw et al., 2010). The gut microbiota have important roles in shaping the immune system as a whole and particularly in models of MS (Ivanov et al., 2009; Ochoa-Reparaz et al., 2009; Berer et al., 2011). Given the extensive interaction between autophagy and different microbes, it would be informative to determine how autophagy and gut microflora interact to influence autoimmune diseases.

Recent evidence showed that autophagy played an important role in pancreatic beta cell functions and might modulate glucose homeostasis as a whole (Ebato et al., 2008; Jung et al., 2008). ER stress has an important role in the pathogenesis of diabetes and autophagy plays a role in this process as well (Quan et al., 2012). Since ER stress is involved in insulin resistance (Ozcan et al., 2006), autophagy might also be involved in insulin resistance by modulating ER stress response.

## Level Four: Secretion, Autophagy, and Autoimmunity

Secretion from cells can proceed through two broad pathways: a well-characterized canonical pathway in which proteins with a signal peptide go through ER and Golgi. However, secretion of proteins without a signal peptide proceeds through an ER-Golgi independent pathway. Interestingly, it was proposed that secretion of such proteins might, in part, be mediated by autophagy (Giuliani et al., 2011). Autophagy-mediated secretion of acyl coenzyme A (CoA) binding protein (ACBP), a cytosolic protein without a signal peptide, was reported in yeasts (Duran et al., 2010; Manjithaya et al., 2010) and recent reports also suggested that autophagy is involved in a number of secretory processes in immune and non-immune cells. Autophagy modulates secretory processes in the context of osteoclastic bone formation (DeSelm et al., 2011), from mast cells (Ushio et al., 2011), intestinal Paneth cells (Cadwell et al., 2008), presynaptic neurotransmission (Hernandez et al., 2012), and secretion of IL-1β (Dupont et al., 2011). Though the relationship between autophagy and IL-1β secretion is complicated owing to the fact that autophagy inhibits inflammasome activation (Nakahira et al., 2011), a recent report showed that baseline autophagy inhibits IL-1β secretion whereas induced autophagy increases secretion of IL-1β (Dupont et al., 2011).

Elevated levels of type I interferon, interferon-alpha (IFN-α) being the prototypic one, is the hallmark of SLE and clinical trials are going on with monoclonal antibodies against IFN-α in SLE (Lichtman et al., 2012). Interestingly, autophagy is also involved in type I IFN secretion. Autophagy is required in plasmacytoid dendritic cells (pDCs), a major source of IFN-α, for sensing ssRNA virus and secretion of IFN-α (Lee et al., 2007). Similarly mTOR inhibition has also been shown to reduce IFN-α secretion by pDCs in response to TLR-9 ligands (Cao et al., 2008), though whether this is mediated by autophagy remains to be elucidated. However, in contrast to pDCs which use Toll-like receptor 7 (TLR7) for sensing ssRNA viruses, most other cell types in the body use cytosolic RNA sensors such as RIG-I and MDA-5, belonging to the RLR family, for this purpose. *Atg5* deficiency in MEF has been shown to increase IFN-α secretion in context of viral infections by suppressing RLR signaling (Jounai et al., 2007; Tal et al., 2009). This finding represents a non-canonical role of *Atg5* (Takeshita et al., 2008; Tal et al., 2009). Non-canonical autophagy was also shown to mediate IFN-α secretion in response to DNA-immune complex (Henault et al., 2012). Thus, modulation of IFN-α secretion by autophagy pathway might play a role in SLE. In a recent study, analysis of SLE metabolome in serum samples of SLE patients showed increased oxidative stress (Wu et al., 2012). Autophagy deficiency is generally associated with increased oxidative stress secondary to accumulation of damaged mitochondria (Zhou et al., 2011b). On the other hand, autophagy inhibition leads to accumulation of p62 (Mizushima and Komatsu, 2011) which, in turn, activates Nrf2 (nuclear factor erythroid 2-related factor 2) (Komatsu et al., 2007). Nrf2 works as a major player in the oxidative stress response pathway (Kaspar et al., 2009). The effect of modulation of autophagy on oxidative stress of SLE warrants further studies.

It would be interesting to test how autophagy-mediated secretory functions influence autoimmune processes. Though considered to be mediated by adaptive immune cells, autoimmune processes, as in MS, are influenced by innate immune cells (Gandhi et al., 2010). The role of autophagy in secretion might have added significance in cells such as NK cells and neutrophils, which function mainly through secretion and degranulation. Another important area for future exploration would be the role of autophagy in myeloid-derived suppressor cells that suppress T cell function.

## Conclusion

Given the above potential implications of autophagy in autoimmunity, it is rather surprising that there are only few *in vivo* reports on the functional correlation between autophagy and autoimmune diseases. Non-specific autophagy-lysosomal inhibitors, such as chloroquine, have long been used in clinics to treat SLE and rheumatoid arthritis (He et al., 2011). It is essential to understand the complex interplay between autophagy and autoimmunity in order to develop effective and more specific therapeutic strategies. Autophagy might play different roles in an autoimmune disease depending on the cell types involved and thus the ultimate results of pharmacological modulation might depend on the downstream effector involved. Given the paucity of *in vivo* data, it will be important to determine how the findings from animal models translate to human conditions, as pathophysiology of autoimmune diseases vary considerably between humans and lower animals.

## Conflict of Interest Statement

The authors declare that the research was conducted in the absence of any commercial or financial relationships that could be construed as a potential conflict of interest.
